# CD36 is required for human sapovirus propagation

**DOI:** 10.1128/jvi.01325-25

**Published:** 2025-11-05

**Authors:** Tomoichiro Oka, Yuko Okemoto-Nakamura, Hirotaka Takagi

**Affiliations:** 1 Department of Virology II, Japan Institute for Health Security, National Institute of Infectious Diseases 13511, Tokyo, Japan; 2 Division of Biomedical Food Research, National Institute of Health Sciences, Kanagawa, Japan; 3 Department of Biochemistry and Cell Biology, National Institute of Infectious Diseases, Japan Institute for Health Security 739298 , Tokyo, Japan; 4 Research Center for Biosafety, Laboratory Animal and Pathogen Bank, National Institute of Infectious Diseases, Japan Institute for Health Security 739298 , Tokyo, Japan; University of Michigan Medical School, Ann Arbor, Michigan, USA

**Keywords:** human sapovirus, CRISPR/Cas9 based genome-wide knockout screening, CD36, lentiviral vector, conventional cell lines, conjugated bile acids, antigen ELISA

## Abstract

Human sapoviruses (HuSaVs), which cause acute gastroenteritis, are highly diverse. Fifteen HuSaV genotype strains (GI.1-7, GII.1-5, -8, and GV.1-2) were efficiently propagated in the human duodenum-derived cell line HuTu80 when supplemented with conjugated bile acid (T. Oka, T.-C. Li, K. Yonemitsu, Y. Ami, et al., J Virol 98:e00639-24, 2024, https://doi.org/10.1128/jvi.00639-24). However, the host cellular factors involved in HuSaV infection and propagation remain unidentified. Using a knockout approach, we newly identified and confirmed that CD36 is a critical cellular protein for the propagation of all 15 HuSaV genotypes tested in HuTu80 cells. When the CD36 gene was re-expressed in CD36 gene knockout HuTu80 cells, all HuSaV genotype strains, except for GI.6 and GII.3, recovered their viral propagation ability. To further demonstrate that human CD36 is critical for HuSaV propagation, we expressed human CD36 in HuSaV-insensitive non-human origin cells. Of the 15 HuSaV genotype strains tested, 10 from GI, GII, and GV (GI.3, -5, -6, GII.1-3, -5, -8, GV.1, and GV.2) were propagated in Chinese Hamster Ovary-K1 cells, and six from GII (GII.1-5, -8) were propagated in Vero cells. Although there were differences in the propagation ability between the genotypes tested, at least one of the three cell lines engineered to express the human CD36 gene acquired HuSaV propagation potential, confirming that human CD36 is an essential cellular factor for HuSaV propagation.

**IMPORTANCE:**

The host cellular factor(s) involved in the infection and propagation of sapovirus, which causes acute gastroenteritis in humans, remain unclear. By using a loss-of-gene function approach with a potential physiological infection site derived from a human cell line clone, which caused a marked cytopathic effect related to human sapovirus (HuSaV) propagation, we identified that CD36 was essential for the propagation of all 15 genotype strains of HuSaV tested. We confirmed different effects on distinct genotypes of HuSaV propagation by the re-expression of human CD36 in HuTu80 cells and human CD36 introduced into two non-human HuSaV-insensitive cells. This finding is an important step toward understanding common and genogroup/genotype-specific HuSaV propagation mechanisms, and the development of methods to control this highly contagious virus, including research on the development of anti-viral drugs and the establishment of HuSaV-susceptible animal models in the future.

## INTRODUCTION

Similar to human noroviruses, human sapoviruses (HuSaVs) are transmitted orally and cause acute gastroenteritis in individuals of all ages. HuSaVs can be transmitted via contact between humans, and food-borne and water-borne HuSaV suspected outbreaks have also been reported (1–9). Currently, no vaccine or medicine is available for the treatment of HuSaV, and therefore, it is difficult to control.

HuSaVs are genetically and antigenically diverse and have been classified into four genogroups (GI, GII, GIV, and GV) consisting of at least 18 genotypes (GI.1-7, GII.1-8, GIV.1, GV.1, and GV.2) (10). These classifications are based on the sequences of the major capsid protein (VP1), which constitutes the surface structure of viral particles (10–14). Recently, we successfully propagated and serially passaged HuSaV strains of 15 genotypes using the high-passage-number human duodenal cell line HuTu80 supplemented with conjugated bile acids (12, 15). HuSaV propagation in Caco-2 cells and their derivative was recently reported (16). HuSaV replication has also been reported in human small intestine enteroids (17) and in induced pluripotent stem (iPS)-derived human intestinal epithelial cells (18). However, the essential cellular factor for HuSaV propagation remains unknown.

In this study, we identified a cellular protein factor that was critical for HuSaV propagation.

## RESULTS

### Candidate host factors for HuSaV propagation in HuTu80 cells

**Fig 1:**
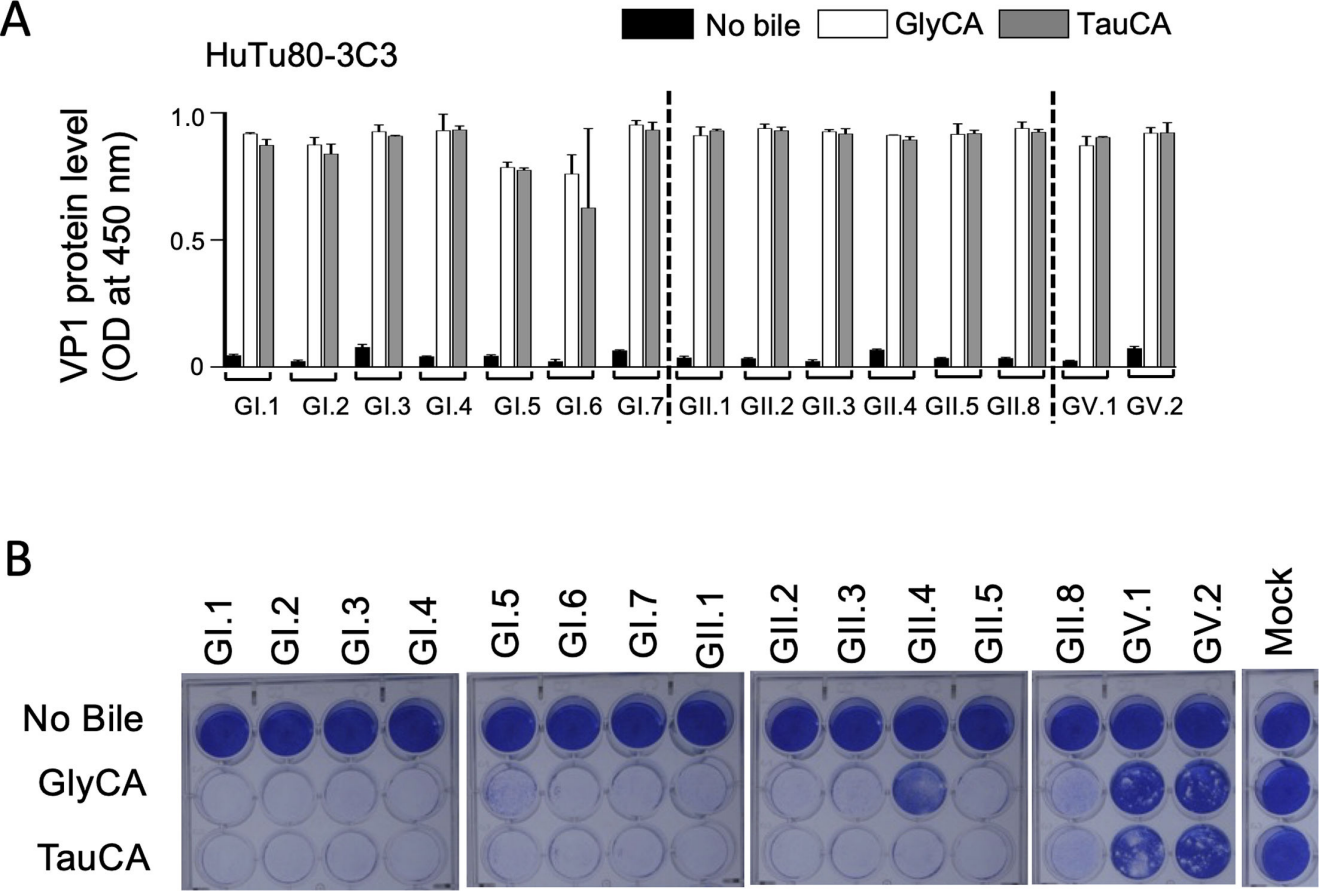
HuSaV propagation in HuTu80-3C3 cells. Propagation (**A**) and cytopathic effects (**B**) of the human sapovirus 15 genotype on HuTu80-3C3 cells. Virus propagation was determined by measuring VP1 protein levels using enzyme-linked immunosorbent assay (ELISA) optical density (OD) values. Data represent the mean of biological duplicates. Error bars indicate the standard deviation.

We identified candidate cellular factors that might be essential for HuSaV propagation by the clustered regularly interspaced short palindromic repeats (CRISPR)/CRISPR associated protein 9 (Cas9) based genome-wide knockout screening of a clone of HuTu80 cells (HuTu80-3C3) that supported the propagation of 15 HuSaV genotypes (GI.1-7, GII.1-5, -8, GV.1, and GV.2), when supplemented with conjugated bile acids, sodium glycocholate (GlyCA) or sodium taurocholate (TauCA) (Fig. 1A), similar to that in HuTu80 cells (Fig. S1A) (12). In contrast to HuTu80 cells, the HuSaV GII.4 strain could propagate in HuTu80-3C3 when supplemented with GlyCA (Fig. 1A; Fig. S1A). Most HuSaV strains tested, other than GV strains, had a marked cytopathic effect (CPE) when supplemented with bile acids (Fig. 1B). Among them, two GI strains, GI.1 and GI.6, and two GII strains, GII.2 and GII.3, were selected as representative strains. Although GlyCA and TauCA supported these HuSaV propagation (Fig. 1A), TauCA was selected for its lower cost (1/5).

**Fig 2:**
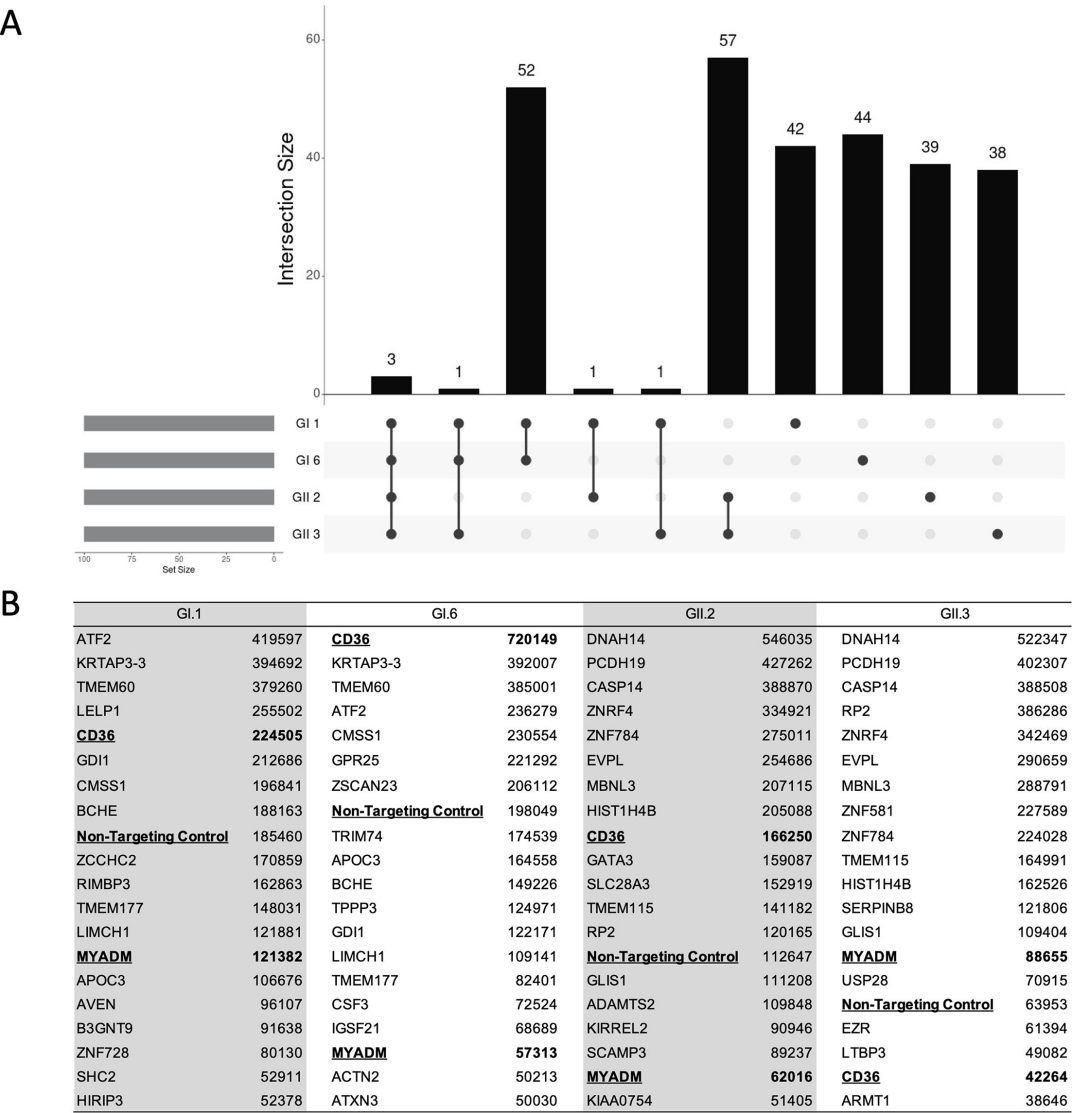
Identification of candidate genes essential for HuSaV propagation. Top hit reads from sapovirus propagation-resistant HuTu80-3C3 cells against four tested human sapovirus genotypes, GI.1, GI.6, GII.2, and GII.3. (**A**) UpSet plot with a TOP 100 gene set. (**B**) List of genes and reads of the TOP 20 gene set.

Then, the number of reads of single guide (sg)RNAs for knockout genes from cells that survived repeated inoculation with these HuSaVs was counted. Upset plot analysis of the top 100 genes revealed guide RNAs targeting three genes were consistently detected in four HuSaV strains (GI.1, GI.6, GII.2, or GII.3) resistant cells (Fig. 2A). These guide RNAs targeted CD36, myeloid-associated differentiation marker (MYADM), and a non-targeting control (Fig. 2B).

The number of sgRNA reads in HuSaV-resistant HuTu80 cells was higher for the CD36 gene than for the MYADM gene except for HuSaV GII.3 (Fig. 2B). In this study, we mainly focused on CD36 (see Discussion).

### Knockout of CD36 abolishes HuSaV propagation ability in HuTu80 cells

**Fig 3:**
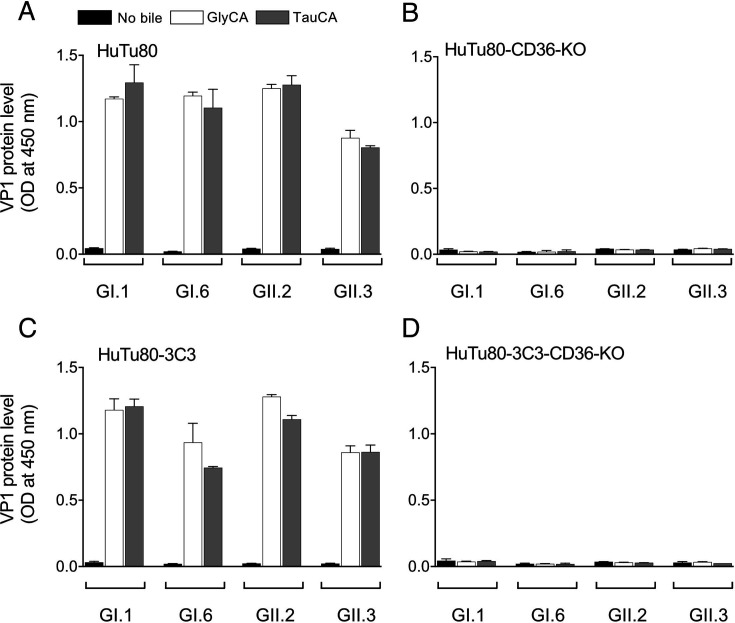
Propagation ability of HuSaV in HuTu80, HuTu80-CD36-KO, HuTu80-3C3, and HuTu80-3C3-CD36-KO cells. Propagation of the HuSaV GI.1, GI.6, GII.2, and GII.3 in HuTu80 (**A**), HuTu80-CD36-KO (**B**), HuTu80-3C3 (**C**), and HuTu80-3C3-CD36-KO (**D**) cells. ELISA OD values are shown. Data represent the mean of biological duplicates, and error bars represent standard deviations.

**Fig 4:**
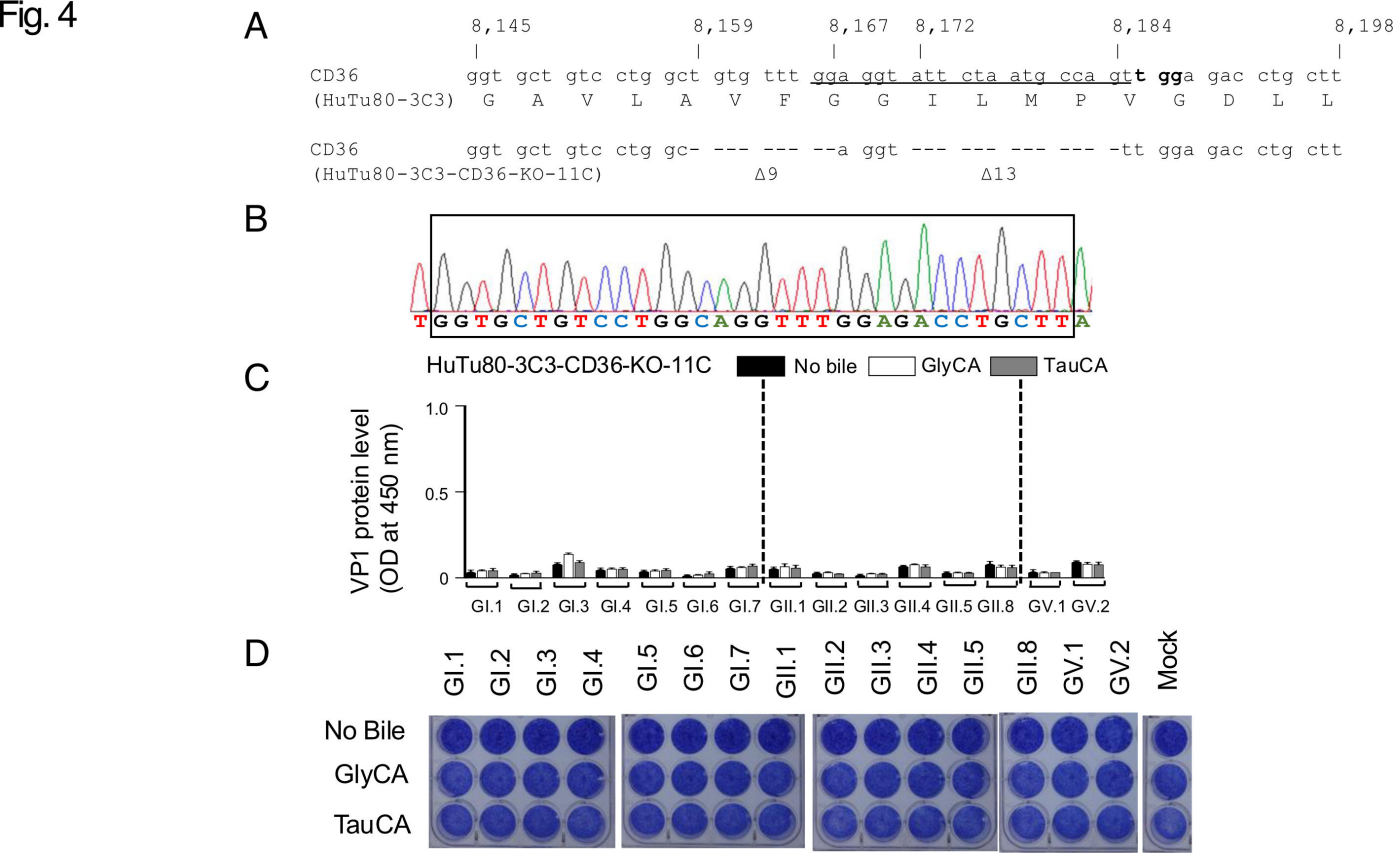
Identification of genomic deletions in HuTu80-3C3 clone 11C cells edited using the CRISPR/Cas9 system and the knockout effect on HuSaV propagation. (**A**) Schematic representation of the target genomic region. The CRISPR/Cas9 target nucleotide sequence and their translated amino acid sequence within exon 3 of the human CD36 gene (19) in HuTu80-3C3 cells are shown in the upper panel, including the 20-nucleotide gRNA sequence (underlined) and the adjacent protospacer adjacent motif sequence (indicated in bold). CRISPR/Cas9-induced deletions in HuTu80-3C3 human CD36 KO clone 11C cells (HuTu80-3C3-CD36-KO-11C) included a 9-base pair deletion from position 8,159 to 8,167 and a 13-base pair deletion from position 8,172 to 8,184. The sequences of these deletions are shown in the lower panel. The numbering of the positions corresponds to the NCBI reference sequence: NG_008192.1. (**B**) Sanger sequencing chromatogram of the edited region. The chromatogram shows the junction sequences, confirming the precise deletions. (**C**) CD36 gene knockout effects on the propagation of 15 HuSaV genotypes. Data represent the mean of biological duplicates, and error bars represent standard deviations. (**D**) The CD36 gene knockout effects on the cytopathic ability related to the propagation of 15 HuSaV genotypes.

To evaluate whether CD36 affects the propagation ability of HuSaV, we knocked out (KO) the CD36 gene in HuTu80-3C3, as well as in a parent HuTu80 cells. GI.1, GI.6, GII.2, and GII.3 HuSaV strains used in the CRISPR screening propagated in HuTu80 and HuTu80-3C3 cells when supplemented with GlyCA or TauCA, but not in HuTu80-CD36-KO and HuTu80-3C3-CD36-KO (Fig. 3). Gene modifications in HuTu80-3C3 CD36 knockout clone 11C cells (HuTu80-3C3-CD36-KO-11C) were confirmed by genome sequencing (Fig. 4A and B). There was no increase in HuSaV VP1 protein levels in the culture supernatant and no obvious CPE was observed for any of the 15 HuSaV genotypes used to infect the HuTu80-3C3-CD36-KO-11C cells with or without bile acid supplementation (Fig. 4C and D).

Therefore, we concluded that CD36 is required for HuSaV propagation.

### Restoration of HuSaV propagation capacity by the re-expression of human CD36 in knockout HuTu80 cells

**Fig 5:**
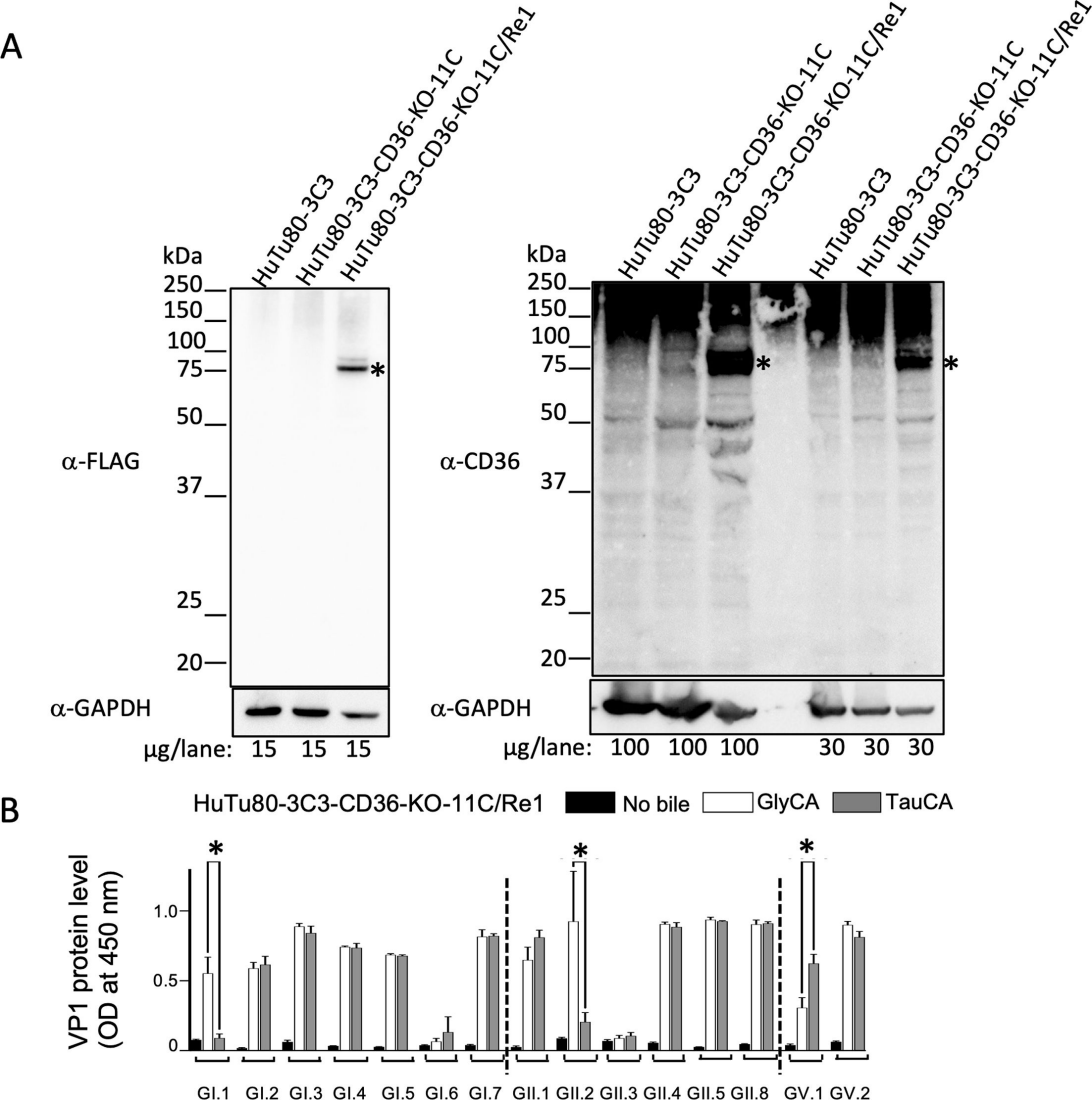
Human CD36 re-expression allows the propagation of HuSaV in CD36 knockout HuTu80-3C3 clone cells. (**A**) Confirmation of protein CD36 expression in HuTu80-3C3 cells, HuTu80-3C3 human CD36 KO clone 11C cells (HuTu80-3C3-CD36-KO), and human CD36 isoform 1 gene reintroduced HuTu80-3C3 human CD36 KO clone 11C cells (HuTu80-3C3-CD36-KO/Re1). Cell lysates were prepared, and the amounts of protein indicated in the bottom panel were subjected to western blotting with anti-FLAG, anti-CD36, or anti-GAPDH antibodies. The protein band corresponding to the putative CD36 is labeled with an asterisk. (**B**) The effect of human CD36 re-expression on the propagation of 15 HuSaV genotypes in the CD36 isoform 1 gene reintroduced HuTu80-3C3 CD36 KO clone 11C cells (HuTu80-3C3-CD36-KO/Re1). Data represent the mean of biological duplicates, and error bars represent standard deviations. Asterisk denotes significance at *P* < 0.001 of the VP1 level between GlyCA and TauCA supplementation within each HuSaV genotype.

To eliminate the possibility of the off-target effects of CRISPR/Cas9, we confirmed that the reintroduction of the human CD36 gene restored HuSaV propagation ability. To achieve this, a synthetic CRISPR/Cas9 recognition sequence-synonymous mutated CD36 isoform 1 gene with a tag coding sequence added to the C-terminus of the expressed protein was introduced into HuTu80-3C3-CD36-KO-11C cells. Although endogenous CD36 expression in HuTu80-3C3 cells could not be detected with the anti-CD36 antibody used in this study (Fig. 5A). The expression of the introduced CD36 was detectable by anti-FLAG and anti-CD36 antibodies in human CD36 isoform 1 gene-introduced HuTu80-3C3-CD36-11C cells (HuTu80-3C3-CD36-KO-11C/Re1) (Fig. 5A). CD36 gene expression was similarly confirmed in HuTu80 3C3 cells with or without bile acid supplementation by reverse transcription-PCR (RT-PCR), but was not detected in HuTu80-3C3-CD36-KO-11C cells (Fig. S2). This suggests the undetectability of endogenous CD36 in HuTu80 3C3 cells at the protein level is likely due to the insufficient sensitivity of the anti-CD36 antibody to detect endogenous levels of CD36, because a protein band corresponding to CD36 was detected in HuTu80-3C3-CD36-KO-11C/Re1 cells (Fig. 5A).

Viral propagation, determined by an increase in HuSaV VP1 protein levels in the culture supernatant, in HuTu80-3C3-CD36-KO-11C/Re1 cells was observed for the 15 genotypes tested under bile acid supplemented conditions except for GI.6 and GII.3 (Fig. 5B). VP1 signal for GI.1 increased only with GlyCA, and for GII.2, the mean optical density (OD) of VP1 was GlyCA > TauCA. For GV.1, the mean OD value was TauCA > GlyCA (Fig. 5B), showing HuSaV propagation varied by bile acid type. Re-expression of human CD36 in knockout HuTu80 cells restored HuSaV propagation capacity in 13 HuSaV genotypes.

### Acquisition of viral propagation potential by introduction of the human CD36 gene to HuSaV-insensitive non-human origin cells

Chinese hamster ovary (CHO) and Vero cells transduced with a control lentivirus vector encoding mCherry did not support the virus propagation ability of the 15 HuSaV strains tested with or without bile acid supplementation (Fig. S1C and D). Based on these results, they were used as representative HuSaV-insensitive non-human origin cells for human CD36 complementation.

**Fig 6:**
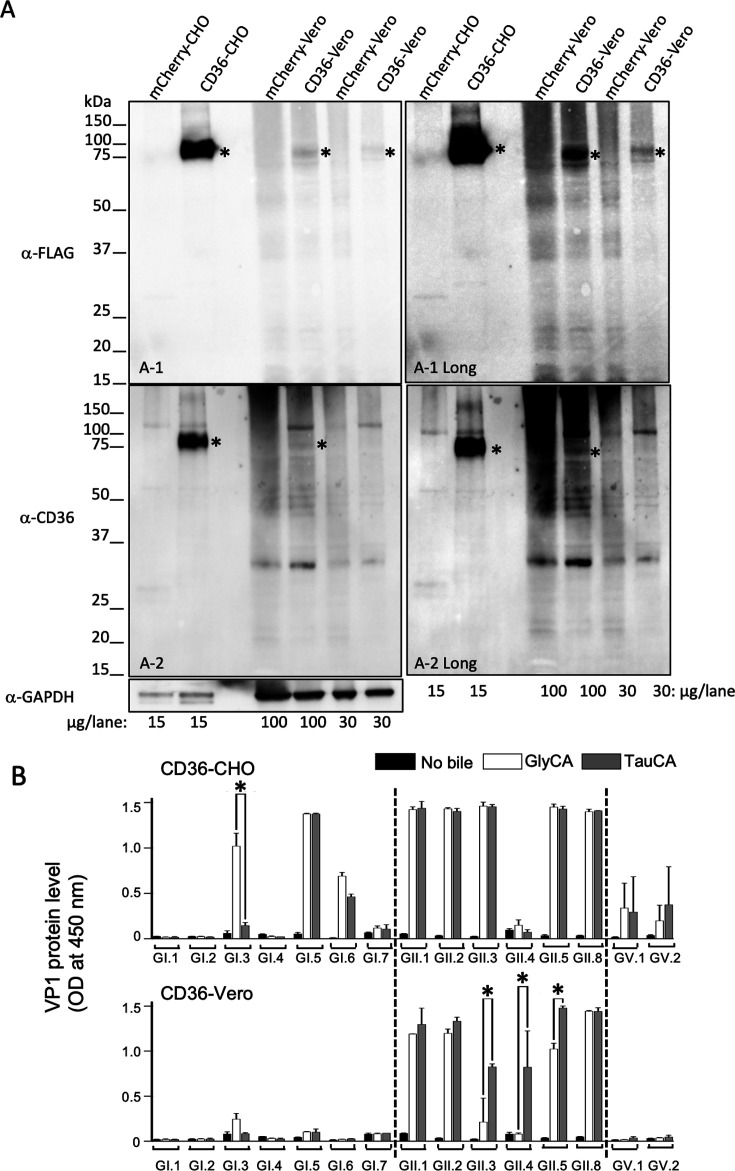
Non-HuSaV susceptible CHO and Vero cells acquire HuSaV propagation ability after human CD36 gene introduction. Confirmation of transduced CD36 protein expression in non-HuSaV susceptible CHO and Vero cells. (**A**) Cell lysates were prepared from CHO and Vero cells, both of which expressed either mCherry or human CD36, and western blotting was performed with an anti-FLAG antibody (panel A-1), anti-CD36 antibody (panel A-2), or anti-GAPDH antibody. For the anti-FLAG and anti-CD36 antibodies, longer exposure images are also shown (A-1 Long and A-2 Long, respectively). The protein band corresponding to the putative CD36 is labeled with an asterisk. (**B**) The effect of human CD36 expression on the propagation of 15 HuSaV genotypes in CHO or Vero cells. Data represent the mean of biological duplicates, and error bars represent standard deviations. Asterisk denotes significance at *P* < 0.001 of the VP1 level between GlyCA and TauCA supplementation within each HuSaV genotype.

CHO and Vero cells were transduced with the human CD36 isoform 1 gene and the production of CD36 was confirmed by western blotting using anti-FLAG and anti-human CD36 antibodies (Fig. 6A). In CHO cells transduced with the human CD36 gene (CD36-CHO), 10 of the 15 HuSaV genotypes (GI.3, GI.5, GI.6, GII.1, GII.2, GII.3, GII.5, GII.8, GV.1, GV.2) showed HuSaV propagation under bile acid-supplemented conditions, as demonstrated by an increase in HuSaV VP1 protein in the culture supernatant. The VP1 OD value of HuSaV GI.3 was GlyCA > TauCA (Fig. 6B, upper panel).

In contrast, in Vero cells transduced with the human CD36 isoform 1 gene (CD36-Vero), only the six HuSaV GII strains (GII.1, GII.2, GII.3, GII.4, GII.5, and GII.8) showed viral propagation under bile acid-supplemented conditions, as demonstrated by an increase in HuSaV VP1 protein in the culture supernatant. HuSaV GII.4 propagated only with TauCA supplementation. VP1 OD value of HuSaV GII.3 and GII.5 was TauCA > GlyCA supplementation (Fig. 6B, lower panel). In western blot analysis using Vero cells, the production of the introduced human CD36 was detectable with the anti-FLAG antibody, but no clear signal was observed with the anti-CD36 antibody even after increasing the amounts of cell lysate (Fig. 6A). The difference in transduced CD36 expression between CHO and Vero cells may be partially related to the different puromycin-resistant concentrations (50 and 3.2 µg/mL, respectively). We hypothesize that the observed difference in HuSaV propagation ability between human CD36-introduced CHO and Vero cells may be partially related to the differences in human CD36 production levels between these two cell lines.

Expression of human CD36 results in the acquisition of HuSaV propagation ability in 11 HuSaV genotypes to HuSaV-insensitive non-human origin cells.

### Different effects on distinct genotypes of HuSaV propagation between human CD36 gene knockout and expression

The knockout of the human CD36 gene prevented the propagation of all 15 HuSaV genotypes in HuTu80-3C3-CD36-KO-11C cells (Fig. 4C). Furthermore, HuSaV propagation was confirmed under the conjugated bile acid supplementation condition in at least one of the following (i) human CD36 isoform 1 gene-introduced HuTu80-3C3-CD36 gene KO clone 11C cells (HuTu80-3C3-CD36-KO-11C/Re1) (Fig. 5B), (ii) CHO cells with the introduced human isoform 1 CD36 gene (CD36-CHO), or (iii) Vero cells with the introduced human isoform 1 CD36 gene (CD36-Vero) (Fig. 6B).

Of note, the HuSaV GI.6 and GII.3 strains did not re-acquire viral propagation potential in HuTu80-3C3-CD36-11C-KO/Re1 (Fig. 5B). However, these two HuSaV genotype strains were able to propagate in CD36-CHO (Fig. 6B, upper panel). The HuSaV GII.4 strain, which did not propagate in CD36-CHO cells, was propagated in CD36-Vero cells and HuTu80-3C3-CD36-KO-11C/Re1 (Fig. 6B and 5B).

Based on these results, we conclude that human CD36 is an essential cellular factor common to all 15 HuSaV genotypes that propagate in HuTu80 cells. Conjugated bile acids, GlyCA and/or TauCA, were necessary supplements for HuSaV propagation under all of the tested conditions.

## DISCUSSION

We previously reported HuSaV and human parechovirus propagation in HuTu80 cells (15, 20) and identified MYADM as an essential factor for human parechovirus propagation (21). The MYADM knockout did not eliminate HuSaV propagation ability using 15 genotypes in HuTu80 cells (Fig. S1B) than in the HuTu80 cells (Fig. S1A). Based on these data, we searched for other cellular factors essential for HuSaV propagation by CRISPR screening, with HuTu80 3C3 clones showing clear CPE upon HuSaV propagation (Fig. 1B) and CD36 emerging as the top candidate genes for the four HuSaV genotypes (Fig. 2B).

CD36 is expressed in various human tissues, including the gastrointestinal tract (https://www.proteinatlas.org/ENSG00000135218-CD36/tissue). CD36 RNA expression has been reported in HuTu80 cells (https://www.proteinatlas.org/ENSG00000135218-CD36/cell+line). We confirmed CD36 gene expression in HuTu80-3C3 cells using RT-PCR (Fig. S2).

In our previous studies, we used enzyme-linked immunosorbent assay (ELISA) and reverse transcription-quantitative PCR (RT-qPCR) as HuSaV propagation indicators (12, 15) and demonstrated that the results of these methods were consistent. In this study, we used Ag-ELISA for all experiments because of its simplicity. The inoculated HuSaV had a low ELISA OD value (approximately 0.1 or lower). After virus inoculation, we maintained the culture for 10 days post-inoculation (dpi) without washing and measured VP1 levels in the supernatant to determine increases compared to the input levels. This protocol was previously used (12) and has advantages over RT-qPCR, which detects input virus RNA and requires washing steps that leave substantial signals, necessitating comparisons between 1 and 10 dpi (12, 15).

After CRISPR screening with HuTu80-3C3, we introduced CD36 targeting guide RNA into parent HuTu80 and HuTu80-3C3 cells. In both lines, the four HuSaV genotype strains showed no increase in the VP1 signal under bile acid conditions, unlike HuTu80 and HuTu80-3C3 cells (Fig. 3). Loss of HuSaV propagation was further confirmed in four HuTu80-3C3-CD36-KO clones using HuSaV GII.2 inoculation (Fig. S3). Then, we confirmed that the 15 HuSaV genotypes did not propagate in HuTu80-3C3-CD36-KO-11C cells (Fig. 4C).

Lentiviral constructs and CRISPR machinery for MYADM and CD36 knockout were identical, except for the guide RNA sequence, and HuSaV propagation was lost only in CD36 KO cells. We concluded that CD36 was essential for HuSaV propagation in HuTu80 and HuTu80-3C3 cells.

Restoration of HuSaV propagation capacity was confirmed in 13 among 15 genotypes by the re-expression of human CD36 in HuTu80-CD36-KO-11C cells (Fig. 5B). Introducing the CD36 isoform 1 into four HuTu80-3C3-CD36-KO clones also enabled HuSaV propagation (Fig. S3). GII.2 strain was used because it had the highest VP1 signal in the HuTu80-3C3-CD36-KO-11C/Re1 among the four HuSaV genotypes used for CRISPR screening (Fig. 5B). Although the HuSaV GII.2 VP1 OD values in 3C3 were similar to those of GlyCA or TauCA (Fig. 1; Fig. S3), all HuTu80 3C3-CD36-KO-clone cell lines with CD36 isoform 1 expression showed greater HuSaV VP1 OD values for GlyCA than for TauCA (Fig. 5B; Fig. S3). This indicates that, unlike HuTu80-3C3 cells with endogenous CD36, the bile acid type in HuTu80-3C3-CD36-KO clone cells with CD36 isoform 1 expression affected HuSaV propagation.

To investigate whether CD36 alone is sufficient to confer HuSaV propagation ability, CHO and Vero cells were used as representative HuSaV-insensitive cells of non-human origin (Fig. S1C and D), which were used in our previous recombinant protein expression studies (22, 23). Acquisition of HuSaV propagation capacity was confirmed in 11 of the 15 HuSaV genotype strains (Fig. 6B).

Our data support that human CD36 is a key cellular factor enabling HuSaV propagation in the tested cells (Fig. S3; Fig. 5B and 6B).

Human CD36 (isoform 1), a membrane glycoprotein with two transmembrane domains of 472 amino acids, is expressed in various tissues and cells, involved in immunity, metabolism, and angiogenesis. Its ligands include thrombospondin, collagen, and malaria-infected erythrocytes, diverse ligands without structural similarity. It functions in recognition and phagocytosis of foreign substances by monocytes and macrophages, and in the uptake of long-chain fatty acids. The binding of CD36 to these ligands activates intracellular signaling pathways, including Src family kinases, MAP kinases, and PI3K/Akt pathways, altering cellular functions and responses (24–27).

In addition to the 472 amino acid CD36 isoform 1, which is mainly expressed, five other isoforms lacking certain regions are present in the NCBI database (https://www.ncbi.nlm.nih.gov/datasets/gene/id/948/products/) (27). We conducted overexpression experiments for CD36 isoform 2 (433 aa in length, ∆ 234–272 aa) and isoform 3 (412 aa in length, ∆ 144–203 aa) in HuTu80-3C3-KO-11C using the same lentiviral backbone for CD36 isoform 1. Using the GII.2 strain, HuTu80-3C3-CD36KO-11C cells expressing these two CD36 isoforms showed no increase in HuSaV VP1 signals with or without bile acids (Fig. S3). The CD36 isoform protein expression in HuTu80-3C3-CD36-KO-11C, and reduction in protein size following deglycosylation treatment were confirmed by western blotting (Fig. S4). These results show that the expression of CD36 genes other than isoform 1 via the lentiviral backbone does not restore HuSaV propagation ability.

The difference in results when using HuTu80-3C3-CD36-KO, CHO, and Vero cells for CD36 isoform 1 overexpression with distinct genotypes of HuSaVs with different bile acids, GlyCA, and TauCA (Fig. 5B and 6B; Fig. S3) is puzzling and suggests a complex mechanism and pathway for HuSaVs propagation, which may be influenced by the expression of CD36 and activity of bile acids, as well as cell origin and cellular factors other than human CD36, depending on the genogroup or genotype strain.

CRISPR/Cas9 screening also identified other sgRNA sequences of genes other than human CD36 in the HuSaV-infected CPE-resistant cell population (Fig. 2B). The same guide RNA was detected in HuSaV GI.1 and 6 (*n* = 52) and GII.2 and 3 (*n* = 57) resistant cells, respectively, in the top 100 (Fig. 2A; Table S1). Specifically, activating transcription factor 2, keratin-associated protein 3-3, and transmembrane protein 60 ranked higher in GI.1- and GI.6-resistant cells, and dynein axonemal heavy chain 14, protocadherin 19, and caspase 14 ranked higher in GII.2- and GII.3-resistant cells in the top 20 (Fig. 2B). Therefore, evaluating the effects of these cellular factors on HuSaV propagation is warranted in the future.

Consistent with previous reports using enteroids or iPS-derived intestinal cells, fucosyltransferase 2, which is essential for the replication of various human norovirus strains and which had no effect on the replication of HuSaVs (17, 18), was not a candidate in our screening. Furthermore, occludin, previously reported to confer sensitivity to porcine sapovirus in non-permissive CHO cells (28), was also not a candidate in our study.

The non-targeting control guide RNA was also ranked among the top three common genes by CRISPR screening, alongside CD36 and MYADM (Fig. 2). We confirmed the knockout effect of these specific protein-coding genes, especially CD36; however, we did not generate HuTu80 cells using the non-targeting control guide RNA. One possible reason for this is that we introduced the lentivirus at a higher multiplicity of infection than anticipated, introduced gene-specific guide RNA and non-targeting control guide RNA into the same cell; however, this has not been confirmed experimentally.

This study had some limitations. First, HuSaV propagation was verified throughout the study by increased VP1 protein levels via Ag-ELISA. Second, we did not resolve the mechanism by which CD36 is involved in HuSaV propagation. Third, the mechanism by which conjugated bile acids, GlyCA, and TauCA are involved in HuSaV propagation, and why they promoted different effects on the tested human CD36-expressed cells and HuSaV genotypes, was not determined.

To partially resolve the first limitation, we performed a median tissue culture infectious dose (TCID_50_) assay with representative samples using a cell-based ELISA, as described in Materials and Methods. Virus titer ranges for HuTu80-3C3 with GlyCA were 4.0–5.5 log_10_ TCID_50_/50 µL for HuSaV GI.1, GI.6, GII.2, GII.3, and 3.75–5.25 log_10_ TCID_50_/50 µL for GI.1, GI.6, GII.2, GII.3 for TauCA, compared with ≤0.5 log_10_ TCID_50_/50 µL for HuTu80-3C3 without bile acid supplementation and HuTu80-3C3-CD36-KO with/without bile acid supplementation, for all four HuSaV genotypes (Fig. S5).

Virus titer ranges of GI.6, GII.2, and GII.3 in CD36-CHO with GlyCA and TauCA were 4.75–5.0; 4.0–4.5 log_10_ TCID_50_/50 µL, compared with GI.1 ≤0.5 log_10_ TCID_50_/50 µL (Fig. S5). CD36-Vero with GlyCA and TauCA showed higher average virus titers (3.0, 4.75) for GII.2 and were ≤0.5–1.5 log_10_ TCID_50_/50 µL for GI.1, GI.6, and GII.3. Virus titers were ≤0.5 log_10_ TCID_50_/50 µL in cell supernatants without bile acid supplementation (Fig. S5). Thus, increased VP1 protein levels measured by Ag-ELISA reflected infectious virus production in the tested samples (Fig. 3C, D, and 6B; Fig. S5). SaV-virion production in non-susceptible cells might be variable, depending on the genotype or bile acid type.

Additional experiments to determine how these two bile acids impact the localization of CD36 and whether CD36 knockout impacts the binding/entry of HuSaVs to cells, as well as whether the CD36 knockout blockade of HuSaV propagation can be bypassed by transfecting HuSaV RNA into cells, might reveal the potential role of CD36 as an entry factor.

Nevertheless, our findings are an important first step toward understanding HuSaV propagation mechanisms and the establishment of methods to control this highly contagious virus by developing anti-HuSaV and HuSaV-susceptible animal models in the future.

## MATERIALS AND METHODS

### Cell lines

Three human cell lines were used for HuSaV culture, including (i) a human duodenum carcinoma-derived cell line (HuTu80 cells; ATCC #HTB-40) and their clone or genetically modified cells; (ii) a Chinese hamster ovary-derived cell line (CHO-K1 cells; JCRB IFO50414); and (iii) a monkey kidney-derived cell line (Vero cells; JCRB0111).

### Cell culture conditions

HuTu80 cells that had undergone a high number of passages (>150) and selected as a clone were designated HuTu80-3C3 and grown in Iscove’s Modified Dulbecco’s medium (FUJIFILM Wako Pure Chemical Corporation, Osaka, Japan) supplemented with 3% heat-inactivated fetal bovine serum (FBS) (Biosera, Kansas City, MO, USA), and antibiotics (100 U/mL penicillin and 100 µg/mL streptomycin (Thermo Fisher Scientific, Waltham, MA, USA) as previously described (12). Human CD36 gene KO HuTu80-3C3, or their clones, were grown under the above conditions supplemented with puromycin (3.1 µg/mL) (Invivogen, San Diego, CA, USA), and CD36 gene KO HuTu80-3C3 clones with the re-expression of the human CD36 gene were grown under the above conditions supplemented with blasticidin (25 µg/mL) (FUJIFILM Wako Pure Chemical Corporation). CHO-K1 cells were grown in Ham’s F12 medium (FUJIFILM Wako Pure Chemical Corporation) supplemented with 5% FBS and antibiotics. Vero cells were grown in Eagle’s minimal essential medium (FUJIFILM Wako Pure Chemical Corporation), supplemented with 5% FBS and antibiotics. mCherry or human CD36-expressing CHO and Vero cells were grown under the above conditions supplemented with puromycin (50 and 3.1 µg/mL, respectively).

### Viruses

HuSaV passage 1 stock (cell culture supernatant) of one of the 15 genotypes (GI.1-AK20, GI.2-FS25, GI.3-D1736, GI.4-Chiba000496, GI.5-D1729, GI.6-Chiba000764, GI.7-D1714, GII.1-Kuma129, GII.2-Kuma130, GII.3-AK11, GII.4-D1739, GII.5-Kashiwa1, GII.8-AK764, GV.1-D3302, and GV.2-NGY1) (12, 14, 15) were used for viral propagation studies.

### Bile acids

GlyCA or TauCA, both purchased from Nacalai Tesque (Kyoto, Japan), were prepared as a 100 mM stock solution in 20% ethanol, filtered through a 0.22 µm filter, and added as supplements to the HuSaV culture medium as described previously (12).

### CRISPR/Cas9 screening to identify candidate genes

HuTu80-3C3 cells cultured in 6-well plates were infected with commercial ready-to-use all-in-one CRISPR/Cas9 based genome-wide human gene-knockout pooled library lentiviruses (Addgene, Watertown, MA, USA, Catalog #73179) based on the Human Brunello CRISPR knockout pooled library (29), which targets 19,114 human genes, with four different target sequences per gene and 1,000 non-targeting control gRNA sequence that do not recognize any sequence in the human genome with a calculated multiplicity of infection of ~1, using TransDux Max Lentivirus Transduction Reagents (System Biosciences, LLC, Palo Alto, CA, USA) at a culture volume of 1.3 mL per well. One milliliter of culture medium was added the following day, and 4 days later, the medium was replaced with 2 mL of medium supplemented with 2 mM TauCA. Then, the lentivirus-transduced HuTu80-3C3 cells were further infected with HuSaV GI.1-AK20, GI.6-Chiba000764, GII.2-Kuma130, or GII.3-AK11 (1 × 10^6^ viral genomic copies/well) and maintained for 10 days. Six plates (a total of 36 wells each) were used for each genotype.

To select HuSaV propagation-resistant HuTu80-3C3 cells, the cells in each well were trypsinized and then inoculated with HuSaV in medium supplemented with TauCA with the same HuSaV genotype strain combination (1 × 10^7-8^ viral genomic copies/well, higher than the initial inoculation) and maintained for 7–10 days. These steps were repeated four times. Genomic DNA was extracted from the surviving cells in each well using the KANEKA Easy DNA Extraction Kit version 2 (KANEKA Corporation, Tokyo, Japan, KN-T110005). Using this genomic DNA, a 160 bp DNA fragment corresponding to the gRNA regions in surviving cells was amplified by PCR using the forward primer (P5) 5′-TTGTGGAAAGGACGAAACACCG-3′ and reverse primer (P7) 5′-CCAATTCCCACTCCTTTCAAGACCT-3′ according to the Addgene protocol using MightyAmp DNA polymerase Ver. 3 (Takara Bio Inc., Shiga, Japan, R076A) under the following conditions: 98°C for 2 min, followed by 40 cycles of a three-step PCR (98°C for 10 s, 60°C for 15 s, and 68°C for 30 s) as previously described (21). PCR products of 160 bp were extracted and pooled along with the infected HuSaV genotypes. The library preparation of HuSaV-resistant cells was performed using the Illumina TruSeq Nano DNA Sample Prep Kit (Illumina, San Diego, CA, USA). NGS sequencing using a NovaSeq 6000 sequencer (Illumina), and data processing, including read count guide RNA sequencing corresponding to the human genes list provided by Addgene, was performed at Macrogen Incorporated (Tokyo, Japan).

The top 100 guide RNA reads of each of the four HuSaV genotypes were analyzed and visualized common candidate genes using Intervene’s Upset plot (https://asntech.shinyapps.io/intervene/) (30, 31).

### Generation of candidate gene knockout HuTu80 cells

Confluent HuTu80 or HuTu80-3C3 cells cultured in 12-well plates were infected with Ready-to-use Human MYADM or CD36 sgRNA CRISPR All-in-One Lentivirus (Human) (Cat No. K1370716 or 155341110603, Applied Biological Materials Inc., Richmond, Canada), using TransDux Max Lentivirus Transduction Reagents (System Biosciences, LLC) with a volume of 1.3 mL per well. One milliliter of culture medium was added the following day, and 4 days later, the cells were trypsinized, spread onto 24-well plates with medium supplemented with serial dilutions of puromycin, and resistant cells with the highest puromycin concentration (3.2 µg/mL) were expanded.

### Confirmation of target gene knockout by genomic sequencing

HuTu80-3C3 CD36 KO cells were subjected to clonal selection to isolate single-cell clones following CRISPR/Cas9-mediated genome editing. Genomic DNA was extracted from the cells using the NucleoSpin Tissue Kit (Takara Bio Inc.) according to the manufacturer’s instructions. The targeted genomic region of human CD36 was amplified by PCR using locus-specific primers (forward: 5′-A CCA GAG CTT GTA GAA ACC ACT-3′; reverse: 5′-A CAT GCA TAC CTG TAG ACA GCT-3′). Similarly, the targeted genomic region of human MYADM was amplified by PCR using locus-specific primers (forward: 5′-A AGA AAA GAA AAC CGA AAG CCC-3′; reverse: 5′-G TAA GCC AC A CAC GCG ATG-3′) from the HuTu80-MYADM KO cells. The PCR products were subsequently purified using the NucleoSpin Gel and PCR Clean-up Kit (Takara Bio Inc.) and analyzed by Sanger sequencing.

### Confirmation of gene expressions by RT-PCR

Confluent HuTu80-3C3 cells or HuTu80-3C3-CD36-KO-11C cells cultured in 12-well plates were treated with 1 mL of medium supplemented with 1 mM GlyCA, 2 mM TauCA, or without bile acids for 24 h. RNA was extracted from the pooled cells from the three wells for each cell and treatment using High-Pure RNA Isolation Kits (Roche Diagnostics, Mannheim, Germany) following the manufacturer’s instructions. cDNA was synthesized as follows: 10 µL of DNase-treated RNA samples were mixed with a 10 µL mixture, including 20 pmol oligo dT_30_ primer, 1 mM dNTPs, 20 units of recombinant RNase inhibitor (Takara Bio Inc.), and 60 units of ReverTra Ace (Toyobo, Osaka, Japan). Reactions were performed at 30°C for 10 min, 42°C for 30 min, and 95°C for 5 min. PCR was performed using 2 µL of cDNA with primers targeting the partial human CD36 gene, forward primer (Human CD36 F1:5′- CTGTGTTTGGAGGTATTCTAATGCCAG-3′), reverse primer (Human CD36 R1: 5′-CCTGTGGATTTTGCACATCAAAGATCCA-3′), or primer set targeting the partial glyceraldehyde-3-phosphate dehydrogenase (GAPDH) gene, forward primer (:5′-CAATGACCCCTTCATTGACC-3′), reverse primer (5′-TTGATTTTGGAGGGATCTCG-3′), using 10 µL of 2G Fast HotStart ReadyMix with dye (KAPA Biosystems, Wilmington, MA, USA) under the following conditions: 95°C for 5 min, followed by 50 cycles of 95°C for 20 s, 58°C for 20 s, and 72°C for 5 s, and a final extension at 72°C for 1 min. Each PCR reaction mixture of 10 µL was directly applied and analyzed by 20 mM Tris-acetate–0.5 mM EDTA buffer-based 2% agarose gel electrophoresis in the presence of FastGene Midori Green Direct (Nippon Genetics), and the gel image was captured with a Gel Doc Go Imaging System (BioRad, Tokyo, Japan).

### Complemented CD36 gene expression in cloned knockout HuTu80-3C3 cells

Confluent HuTu80-3C3-CD36 KO (HuTu80-3C3-CD36-KO) clone 1C, 1E, 10E, or 11C cells cultured in 12-well plates were infected with ready-to-use custom ordered lentivirus, pLV[Exp]-CMV > hCD36-mut(ns)/Myc/FLAG:P2A:Bsd, vector ID VB220531-1120gfv (Vector Builder, Chicago, IL, USA), which carry a CRISPR/Cas9 Guide RNA resistant human CD36 (isoform 1 encoding 472 amino acids (aa) in length: GenBank accession number NM_000072.3) with C-terminus Myc (EQKLISEEDL) and FLAG-tag (DYKDDDDK) coding sequences. To achieve this, the CRISPR Cas9/guide RNA target sequence of human CD36 (NM_001001547) was modified (67-GGtGGaATctTgATGCCtGTcGG-89) (synonymous substitutions are shown in lower case). Then, the cells were treated as described in the section above, selected with blasticidin (25 µg/mL), and designated as human CD36 isoform 1 gene-introduced HuTu80-3C3-CD36 gene KO clone 11C cells (HuTu80-3C3-CD36-KO-1C, -1E, 10E, or 11C/Re1).

Additional experiments expressing CD36 isoforms 2 and 3 (encoding 433 aa [GenBank accession number NM_001289908.1], and 412 aa [NM_001289909.1] in length, respectively) (27) were performed using the ready-to-use custom ordered lentivirus, vector ID VB220821-1074yvq, -1075csd (Vector Builder), with the same vector, including tag, as well as selection marker to CD36 isoform 1 as described above using HuTu80-3C3-CD36-KO-11C cells and designated as HuTu80-3C3-CD36-KO-11C/Re2 and -Re3, respectively.

### Expression of the human CD36 gene in non-susceptible cells

Ready-to-use lentivirus expression of mCherry pLV[Exp]-Puro-EF1A > mCherry, vector ID VB010000-9497fvv (Vector Builder), or human CD36 isoform 1 with C-terminus Myc and FLAG-tag coding sequences pLV[Exp]-CMV > hCD36[NM_001001547.3](ns)/Myc/FLAG:P2A:Puro, vector ID VB220510-1106fne (Vector Builder) were used to infect CHO and Vero cells. Cells were treated as above and selected with the highest puromycin concentrations (50 µg/mL for CHO, 3.2 µg/mL for Vero, respectively), and designated as mCherry-CHO, CD36-CHO, mCherry-Vero, and CD36-Vero cells, respectively.

### Confirmation of viral propagation by Ag-ELISA

To confirm the propagation of HuSaV, a confluent monolayer of HuTu80, HuTu80-MYADM-KO, HuTu80-CD36-KO, HuTu80-3C3, HuTu80-3C3-CD36-KO, HuTu80-3C3-CD36-KO-1C, -1E, -10E, -11C, HuTu80-3C3-CD36-KO-1C/Re1, -1E/Re1, -10E/Re1, HuTu80-3C3-CD36-KO-11C/Re1, -Re2, -Re3, mCherry-CHO, CD36-CHO, mCherry-Vero, and CD36-Vero cells were cultured in 24-well plates. The culture medium was replaced with 0.5 mL of medium supplemented with 1 mM GlyCA, 2 mM TauCA, or without bile acids, before inoculation. Then, 20 µL of each diluted P1 viral stock of the 15 strains of different genotypes (at the inoculated virus quantity at which HuSaV propagation can be reproducibly confirmed in any genotype strain in HuTu80 3C3: approximately 2 × 10^6^ copies of viral RNA per well) was added. The cells were then incubated for 10 days without washing. To assess the propagation of the 15 genotypes of HuSaVs at the protein level, 50 µL of supernatant collected at 10 days post-inoculation was analyzed by the corresponding genotype-targeting traditional sandwich-based Ag-ELISA against the respective genotypes to detect the HuSaV VP1 protein, as described previously (12, 15).

Because the 15 genotypes used in this study have distinctly different antigenicities (12), different pairs of antisera were used for each genotype.

### Western blotting

The expression of human CD36 and GAPDH was analyzed by western blotting as previously described (32) with minor modifications. Briefly, cells were harvested and lysed in RIPA buffer (Nacalai Tesque Inc.) containing protease inhibitors (cOmplete; Roche) and benzonase nuclease (Sigma-Aldrich).

Selected lysates were treated with peptide-N-glycosidase F (PNGase F; New England Biolabs, MT, USA) at 37°C overnight as described (32). Lysates were mixed with NuPAGE LDS Sample Buffer (Thermo Fisher Scientific) containing 100 mM dithiothreitol, heated at 70°C for 10 min, and separated by SDS-PAGE on 4%–12% NuPAGE Bis-Tris Mini Protein Gels (Thermo Fisher Scientific). Proteins were transferred to PVDF membranes (Thermo Fisher Scientific) and blocked with TBS containing 0.05% Tween-20 (TBST) and 5% non-fat dry milk (Merck Millipore) for 1 h at room temperature. Membranes were probed with an anti-CD36 rabbit monoclonal antibody [EPR6573] (Abcam, ab133625, 1:1,000) overnight at 4°C, followed by incubation with HRP-conjugated anti-rabbit IgG secondary antibody (1:1,000; Jackson ImmunoResearch, 711-036-152, West Grove, PA). After detection, membranes were stripped using WB Stripping Solution (Nacalai Tesque, Kyoto, Japan) and reprobed with an anti-FLAG (anti-DDDDK-tag) monoclonal antibody (MBL, M185-3L, 1:1,000), followed by incubation with HRP-conjugated anti-mouse IgG secondary antibody (1:5,000; Jackson ImmunoResearch, 715-036-150). Following detection of FLAG, membranes were stripped again and reprobed with an anti-GAPDH monoclonal antibody (5A12) (FUJIFILM Wako, 014-25523, 1:5,000) as a loading control. Signals were detected using Immobilon Western Chemiluminescent HRP Substrate (Merck) or SuperSignal West Femto or West Pico Atto substrates (Thermo Fisher Scientific) and visualized using an LAS-3000 mini image analyzer (Fujifilm, Tokyo, Japan).

### Cell-based ELISA for virus titer determination

To confirm the production of infective viruses, a cell-based ELISA was performed using several cell culture supernatants as follows. Briefly, 50 µL of viral suspension was serially diluted 10-fold (10^−1^–10^−8^) in medium supplemented with TauCA and then added to HuTu80 cells cultured in 96-well plates (final volume 150 µL/well). Two wells were used for each dilution. The cultures were maintained for 7 days at 37°C in 5% CO_2_. After removing the cell culture supernatant, cells were fixed with 100 µL of 10% formalin in PBS (−) for ~2 h at room temperature. After washing once with water and once with PBS (−), the cells were permeabilized with 50 µL PBS (−) containing 0.1% Triton-X100 and 0.25% casein for 1 h at room temperature. Cells were then washed once with water and once with PBS (−), incubated with 50 µL 3% H_2_O_2_/20% methanol/acetone/PBS (−) for ~2 h at room temperature, washed once with water and once with PBS (−), and then blocked with 200 µL PBS (−) containing 0.25% casein overnight at 4°C.

The plates were washed thrice with PBS (−) containing 0.1% Tween 20 (PBS-T), and cells were incubated with 50 µL rabbit anti-HuSaV GI.1, GI.6, GII.2, or GII.3 VLP antiserum diluted 1:2,000 for 1 h at room temperature. The plates were washed, and 50 µL HRP-conjugated goat pre-absorbed anti-rabbit IgG (H+L) (ROCKLAND Code #611-103-122; diluted 1:5,000) was added. The plates were incubated for 1 h at room temperature and then washed with PBS-T before adding 50 µL/well of 1 mM of the substrate 3,3′,5,5′-tetramethylbenzidine and 0.01% H_2_O_2_ in citrate buffer (pH 3.5) for 30 min at room temperature. The reaction was stopped with the addition of 50 µL/well 1 N H_2_SO_4_, and the OD was measured at 450 nm, with 750 nm as the reference, using a microplate reader. The cut-off line was set as average OD values ±3 SD of HuSaV non-infected wells (*n* = 5), and the TCID_50_ was calculated using the Kärber formula.

### Statistical analysis

Statistical significance analyses were performed using one-way analysis of variance, followed by Tukey’s multiple comparisons test. Statistical significance was set at *P* < 0.001. Each experiment was performed at least twice, and data from one biological duplicate (two wells for each condition in one representative experiment) are presented. Error bars denote the standard deviation. All statistical analyses were performed using GraphPad Prism 9.5.1 (GraphPad Software, Boston, MA, USA).

## Data Availability

The read count data of CRISPR/Cas9 screening supporting the findings of this study are available within the article (Table S1). Further inquiries can be made by reasonable request to the corresponding author.

## SUPPLEMENTAL MATERIAL

Supplemental figures jvi.01325-25-s0001.pdf Figures S1 to S5.

Table S1 jvi.01325-25-s0002.xlsx Aggregated dataset of guide RNA read counts obtained by CRISPR/Cas9 screening.

ASM does not own the copyrights to Supplemental Material that may be linked to, or accessed through, an article. The authors have granted ASM a non-exclusive, world-wide license to publish the Supplemental Material files. Please contact the corresponding author directly for reuse.
