# Supplementary material for: CD36 is required for human sapovirus propagation
Source: J Virol. 2025 Nov 5;99(11):e01325-25. doi: 10.1128/jvi.01325-25 (PMC12646008; doi:10.1128/jvi.01325-25)
Supplement: Supplemental figures — Figures S1 to S5. [file jvi.01325-25-s0001.pdf]

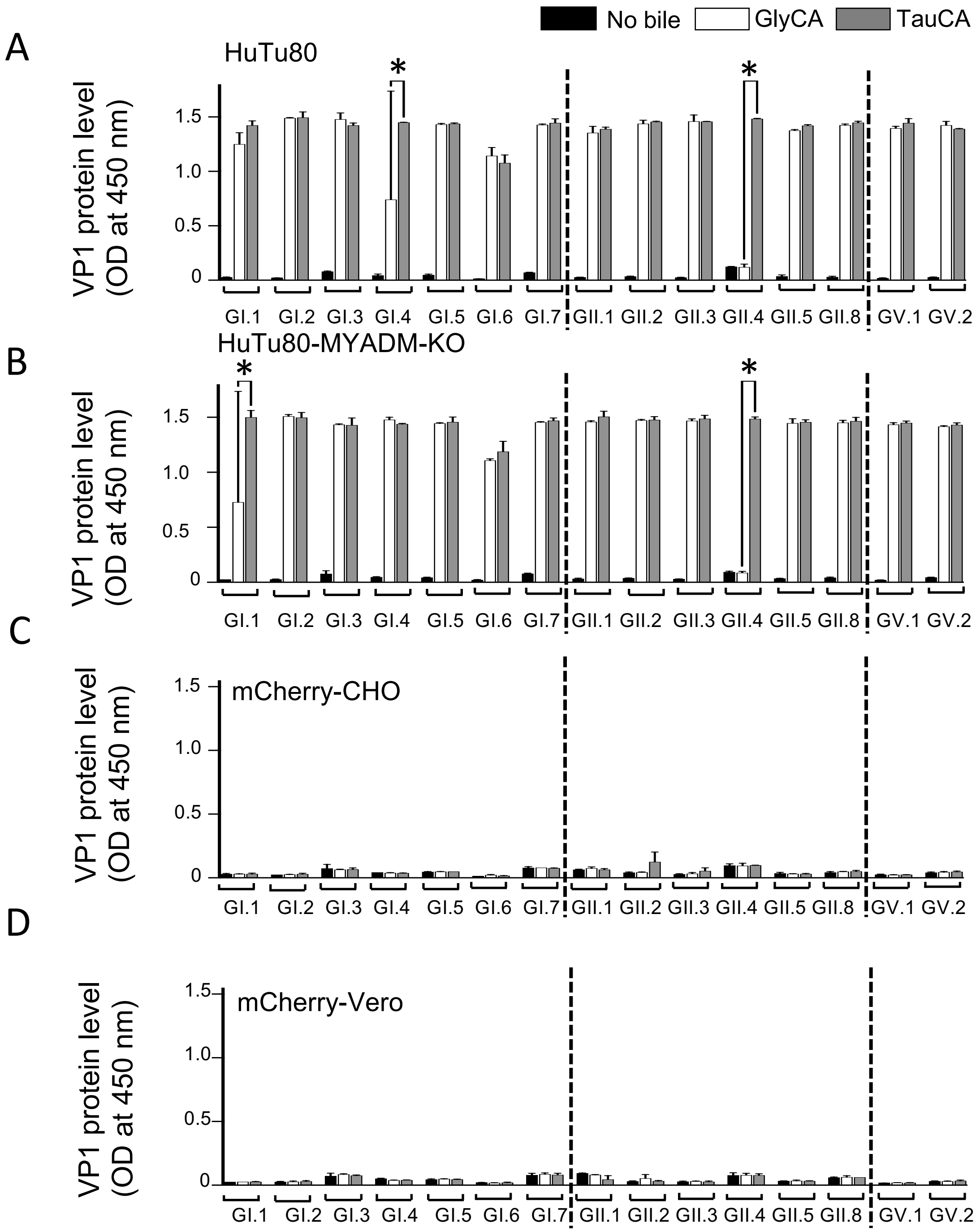

Fig. S1.
Propagation of the 15 HuSaV genotypes in HuTu80 (A), HuTu80-MYADM-KO (B), mCherry-transduced CHO (C), and mCherry-transduced Vero (D) cells. ELISA optical density (OD) values are shown. Data represent the mean of biological duplicates, and error bars represent standard deviations. Asterisk denotes significance at P<0.001 of the VP1 level between GlyCA and TauCA supplementation within each HuSaV genotype.

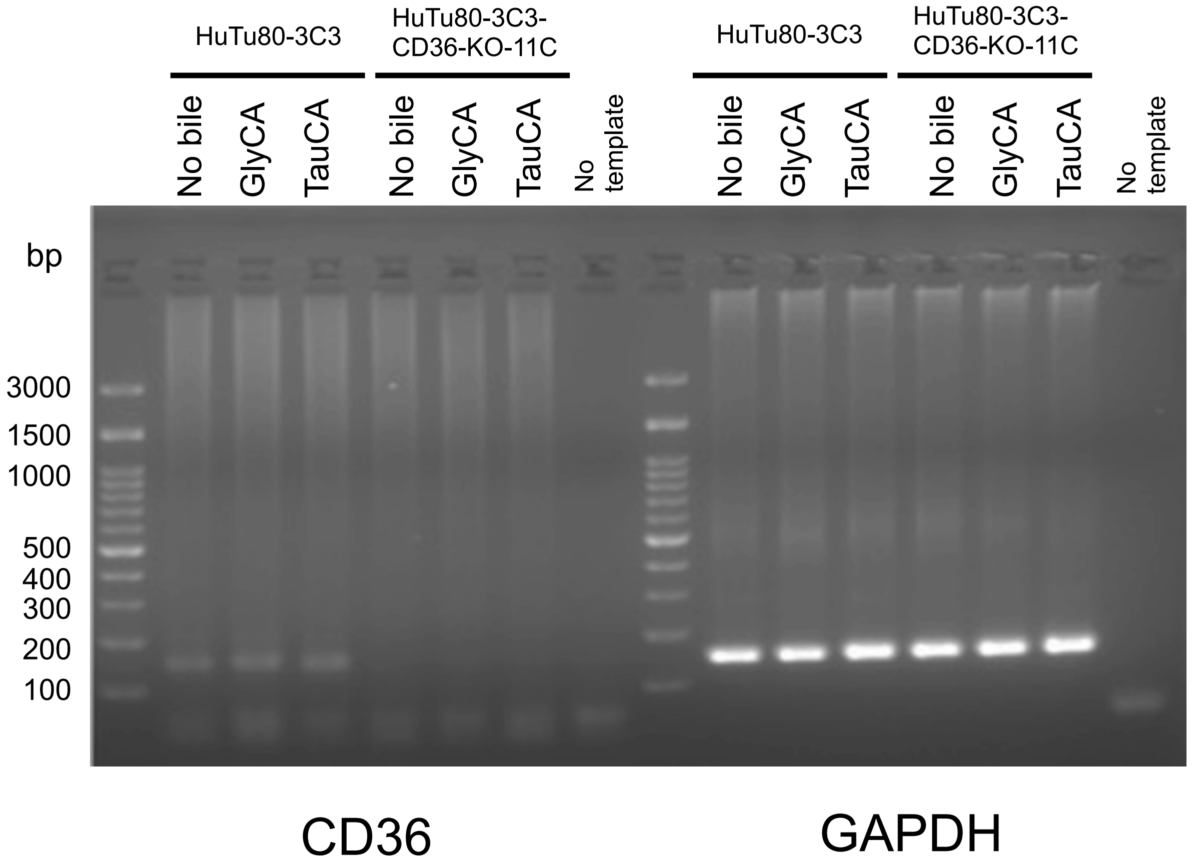

Fig. S2.
Confirmation of CD36 and GAPDH gene expressions in HuTu80-3C3 and HuTu80-3C3-CD36-KO-11C cells with or without GlyCA or TauCA supplementation by RT-PCR.

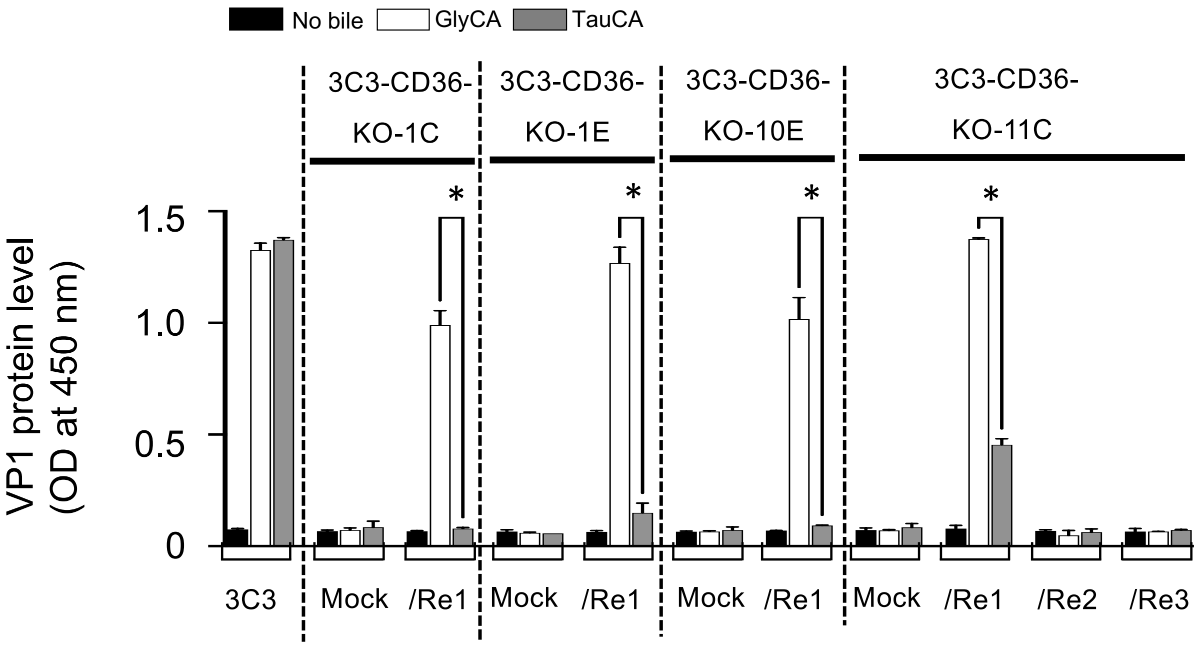

Fig. S3.
Propagation of HuSaV GII.2 in HuTu80-3C3 and a panel of HuTu80-3C3 human CD36 knockout cloned cells, and effects of the re-introduction of a series of human CD36 gene isoforms. The CD36 isoform 1 was re-expressed among four HuTu80-3C3-CD36-KO clones, 1C, 1E, 10E, and 11C (HuTu80-3C3-CD36-KO-1C/Re1, -1E/Re1, 10E/Re1, 11C/Re1). In addition, CD36 isoforms 2 and 3 were expressed in HuTu80-3C3-CD36-KO-11C cells (HuTu80-3C3-CD36-KO-11C/Re2-3). Mock indicates non-treated cells. ELISA optical density (OD) values are shown. Data represent the mean of biological duplicates, and error bars represent standard deviations. Asterisk denotes significance at P<0.001 of the VP1 level between GlyCA and TauCA supplementation within each HuSaV genotype.

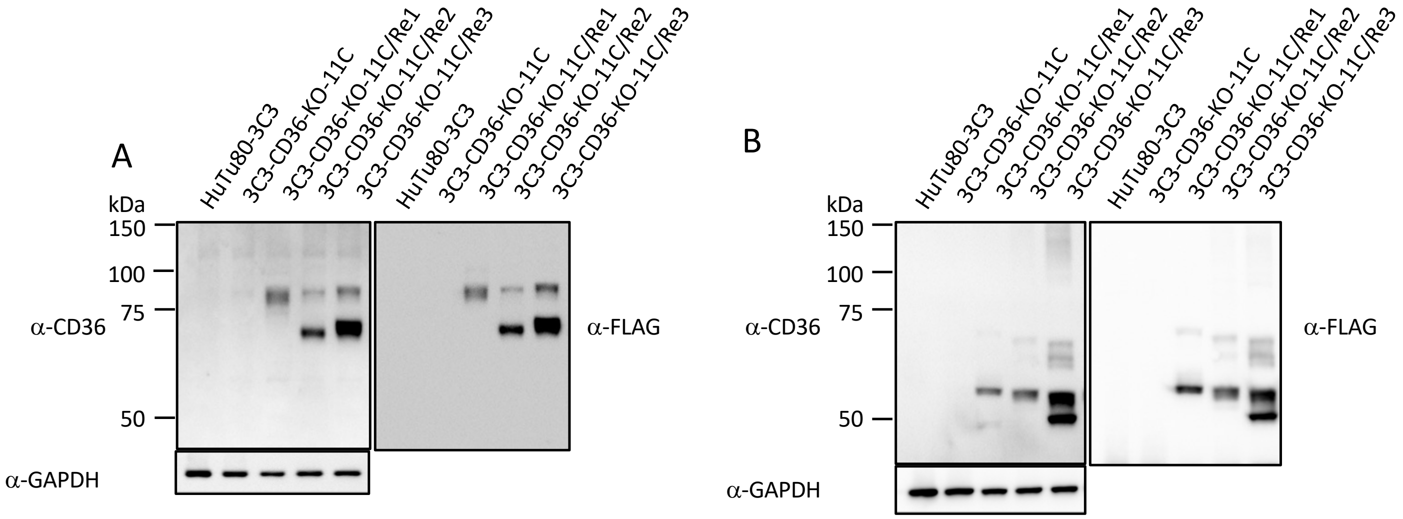

Fig. S4

Confirmation of expression and glycosylation of introduced CD36 isoform 1, 2, and 3 proteins. Cell lysates (10 µg) from parental HuTu80-3C3 cells, HuTu80-3C3-CD36-KO-11C, and HuTu80-3C3-CD36-KO-11C cells expressing FLAG-tagged CD36 isoforms1, 2, and 3 (HuTu80-3C3-CD36-KO-11C/Re1–3) were analyzed by western blotting (A) without PNGase F treatment (B) with PNGase F treatment, and subjected to SDS-PAGE, followed by immunoblotting with an anti-CD36, anti-FLAG or anti-GAPDH antibody.

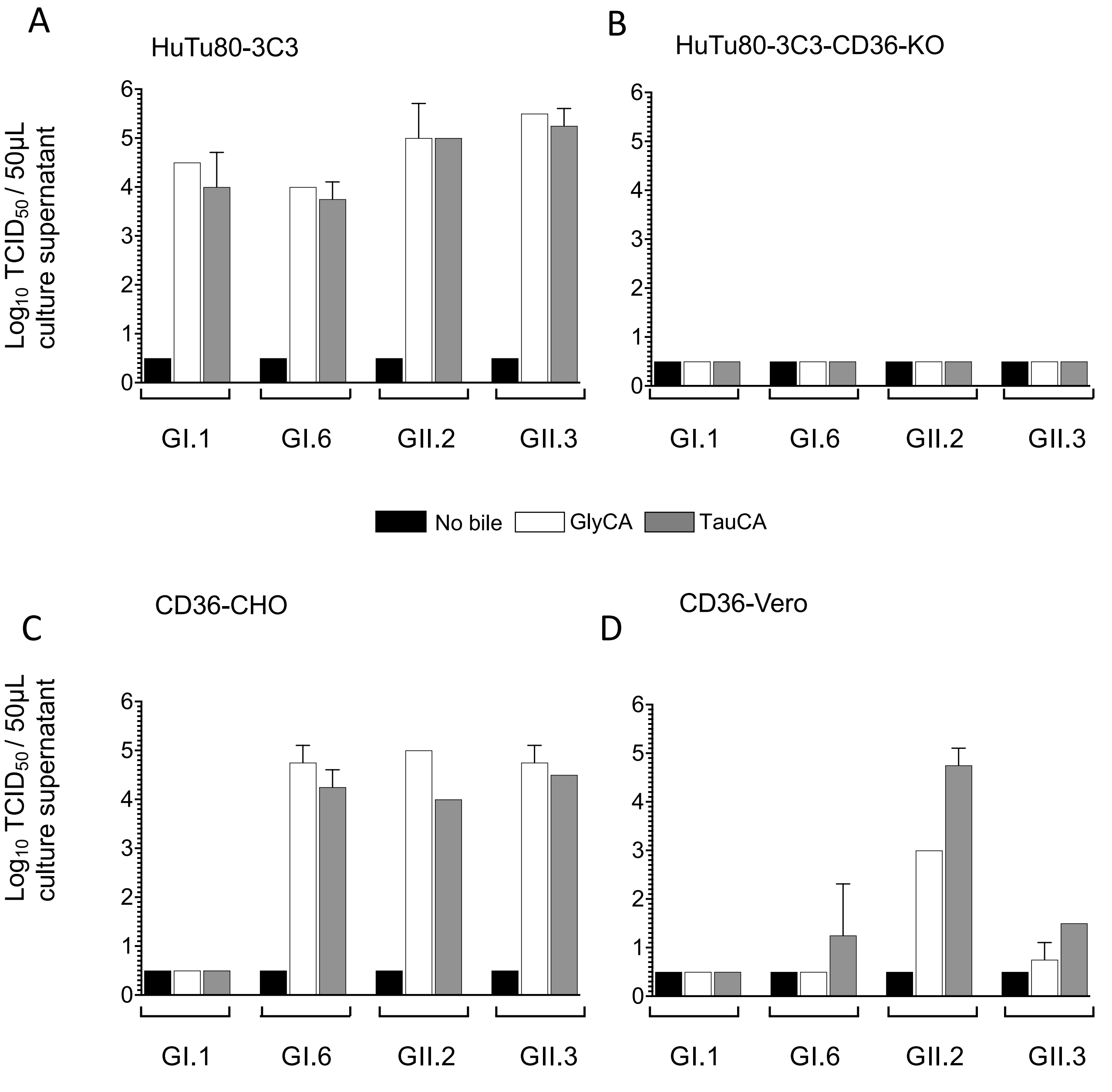

Fig. S5
Virus titer of HuSaV GI.1, GI.6, GII.2, and GII.3 in the 10 dpi supernatant collected from HuTu80-3C3 (A), HuTu80-3C3-CD36-KO (B), CD36-CHO (C) and CD36-Vero (D) with or without GlyCA or TauCA supplementation were shown as Log_{10} $TCID_{50}$ / 50µL. Data represent the mean of biological duplicates, and error bars represent standard deviations.
